# Changes in the Quality Parameters and Antimicrobial Activity of Ozonated Virgin and Pomace Olive Oils Under Different Storage Conditions

**DOI:** 10.3390/foods14060999

**Published:** 2025-03-15

**Authors:** Paula Dominguez Lacueva, Paula Corella Guillamón, María J. Cantalejo Díez

**Affiliations:** Institute for Sustainability & Food Chain Innovation (IS-FOOD), Public University of Navarre (UPNA), Arrosadia Campus, E-31006 Pamplona, Spain; paula.dominguez@unavarra.es (P.D.L.); paula.corella@unavarra.es (P.C.G.)

**Keywords:** ozonated olive oil, pomace olive oil, stability, storage conditions, physicochemical properties, antimicrobial activity, natural preservatives

## Abstract

Ozonated olive oils have emerged as a promising alternative for natural antimicrobial agents in the food industry due to their potential to inhibit microbial growth. However, the stability and effectiveness of these oils under different storage conditions has not been thoroughly explored. This study examines the changes in the physicochemical properties and antimicrobial activity of ozonated virgin olive oil (VOO) and pomace olive oil (POO), stored at 4 °C and 20 °C for 6 months. The peroxide index (PI), acidity index (AI), iodine value (IV), and viscosity (V) were analyzed, along with their antimicrobial activity against *Escherichia coli* (STCC 45), *Pseudomonas aeruginosa* (STCC109), and *Staphylococcus aureus* (STCC 239). The results showed that both oils underwent changes in their physicochemical properties and antimicrobial activity over time. The PI initially increased up to day 30, with VOO reaching a peak value of 741.44 ± 32.16 meq O_2_/kg and POO reaching 1067.23 ± 56.56 meq O_2_/kg, but after this point, it began to decrease in both oils and at both temperatures (4 °C and 20 °C). The acidity index (AI) increased over time, particularly in POO, which reached a final value of 6.32 ± 0.14 mg KOH/g. Both oils showed a reduction in iodine value (IV), and an increase in viscosity (V) over time. In terms of antimicrobial activity, *P. aeruginosa* remained stable with an average inhibition zone of 9.41 ± 0.23 mm, while *E. coli* showed the greatest increase in activity over time, reaching 21.31 ± 4.01 mm in POO at 20 °C. On the other hand, *S. aureus* exhibited the highest average antimicrobial activity, with a mean inhibition diameter of 14.49 ± 0.36 mm, and the largest inhibition zone of Ø = 18.97 ± 1.46 mm observed after 180 days of storage. A Spearman correlation analysis revealed a strong positive relationship (ρ > 0.85, *p* < 0.05) among PI, AI, and the antimicrobial activity with storage duration. This study provides novel insights into the stability of ozonated oils, offering valuable perspectives for their application in the food industry, especially using pomace olive oil, a key by-product in olive oil production.

## 1. Introduction

Ozone (O_3_) is a highly reactive triatomic molecule capable of forming reactive oxygen species (ROS) [1]. Due to its strong antimicrobial activity, ozone has attracted scientific interest for years [2]. However, this reactivity also represents a major limitation, as it makes O_3_ an inherently unstable molecule that can exhibit toxicity to humans. Consequently, ozonated vegetable oils are now the subject of extensive research [3]. Ozone reacts easily with the carbon–carbon double bonds (C=C) of the unsaturated fatty acids present in the triglyceride molecules of vegetable oils. This ozonation reaction was first described by Criegee [4], who demonstrated that the attack of ozone on carbon–carbon double bonds leads to the formation of trioxolane. Upon subsequent decomposition, trioxolane breaks down into carbonyl oxides, ultimately yielding a stable compound known as 1,2,4-trioxolane, or ozonide, which preserves the bactericidal activity of free O_3_. The trioxolane decomposes slowly, generating local oxygen, H_2_O_2_ as reactive oxygen species (ROS), and lipid oxidation products. These compounds act as “ozone messengers” and are the main contributors to the observed biological effects [5] of ozonated vegetable oils. The mechanism of action starts when these compounds bind to glycoproteins, glycolipids, and other amino acids of bacteria to inhibit the enzymatic control system of the bacterial cell, which generates an increase in membrane permeability, allowing ozonides to penetrate the cell causing its lysis and ultimately causing the death of the microorganisms [6].

The antimicrobial effectiveness of ozonated vegetable oils has been repeatedly demonstrated, ranging from their use to treat several infectious illnesses [7,8,9,10] such as skin ulcers, candidiasis, or gingivitis, eliminate *Alternaria alternata* toxins from fresh foods [11], and even extend the shelf life of hamburgers [12] from 3 to 15 days. Studies have shown that ozonated vegetable oils exhibit strong antimicrobial activity against a range of pathogens [13], including *Escherichia coli* [14,15], *Staphylococcus aureus* [15,16], and *Pseudomonas aeruginosa* [17]. In addition, ozonides are known to be liquids or semi-solids at room temperature [18], so it is very important to control their stability under normal storage conditions. However, as Radzimierska-Kaźmierczak et al. [19] states, there is very little scientific literature available about the effects of storage time and temperature on the effectiveness of these oils.

Among the most studied physicochemical properties of ozonated vegetable oils to evaluate their quality and effectiveness, the acidity index (AI, indicating the free fatty acid content), iodine value (IV, assessing the unsaturation grade), and viscosity (V, determining fluidity) are some of the most relevant ones [3]. However, the peroxide index (PI) is considered to be the most important parameter because it indicates the amount of reactive species formed during ozonation; the ones responsible for the biological activity of ozonated vegetable oils. For this reason, the few stability studies that can be found [20,21,22] have primarily focused on the evolution of the peroxide (PI) and acidity indexes (AI) under different storage durations and temperatures. However, these studies overlooked other key quality parameters, such as the iodine value (IV) or viscosity (V), which provide important insights into the structural changes and overall stability of ozonated oils. Additionally, the persistence of antimicrobial activity over time remains largely unexplored. To the best of our knowledge, only Moureu et al. [23] studied the storage-induced changes in the AI, PI, and antimicrobial activity (against *Streptoccocus uberis*) in an ozonated sunflower oil. These knowledge gaps highlight the need for a more comprehensive evaluation of the physicochemical and antimicrobial stability of ozonated vegetable oils during storage.

Various vegetable oils, such as sunflower, coconut, hemp, and krill, have been used for ozonation purposes; however, olive oil is among the most studied one due to its high unsaturated fatty acid content (70–85%) and abundant antioxidants, making it a prime candidate for generating bioactive compounds through ozonation [19]. Similarly, olive pomace, the primary by-product of olive oil production, shares a comparable composition with virgin olive oil (VOO) and holds significant value in various applications. In addition, pomace olive oil (POO) is mainly a source of oleic acid [24], together with a wide variety of bioactive compounds. Given that, POO represents a substantial reduction in expenditure compared to virgin oils, its use for ozonation offers an economically efficient and sustainable approach.

Therefore, the present study aims to evaluate, for the first time, the changes in both, the main quality parameters (PI, AI, IV, and V) and the antimicrobial activity against three bacterial strains (*E. coli*, *P. aeruginosa*, and *S. aureus*) of two ozonated olive oils (virgin olive oil and pomace olive oil) stored at 4 °C and 20 °C for 6 months. This research will contribute, not only to broaden our knowledge about the stability of ozonated vegetable oils under different storage conditions, but also to provide deeper insights about the relationship between the physicochemical parameters and the biological effectiveness of ozonated oils. Additionally, one of the ozonated olive oils under study is derived from pomace, which is considered the main waste product of the olive oil industry. Thus, the reutilization of this residue as a potential antimicrobial agent will contribute to circular economy and sustainability within the food chain.

## 2. Materials and Methods

### 2.1. Samples

This study investigated the effects of storage conditions on two types of olive oils provided by Biosasun S.A. (Navarra, Spain). Virgin olive oil (VOO), obtained directly from olives (Arroniz variety) through mechanical processes, and pomace olive oil (POO), obtained from a second mechanical extraction of the solid residues (pomace) produced after the initial extraction of virgin olive oil in the 2023-24 harvest.

### 2.2. Ozonation and Storage Conditions

**Figure 1:**
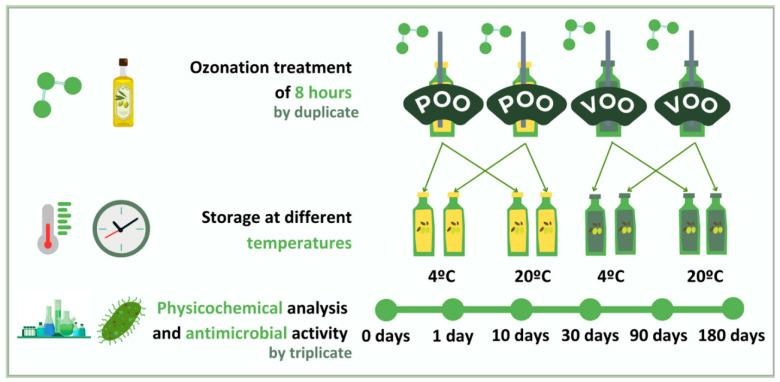
Graphical illustration of experimental design where POO is pomace olive oil and VOO is virgin olive oil.

From 5-litre bottles of virgin olive oil (VOO) and pomace olive oil (POO), random and representative samples of 150 mL of each oil were taken by duplicate (resulting in a total of four bottles). The ozonation treatment was carried out in all samples, for a duration of 8 h at 20 °C. This made it possible to perform the ozonation treatment by duplicate and consider the differences in the ozonation process. Ozone was generated using a EUROZON (170265 Series) ozone generator (Barakaldo, Spain). This equipment produces 6.6 g O_3_/h with a concentration of 55 g O_3_/m^3^ and a flow rate of 2 L min^−1^ of oxygen at 20 °C. These settings were precisely controlled to ensure consistent ozonation conditions. Subsequently, each ozonated oil sample of 150 mL was divided into two bottles, adding up to a total of eight bottles of 75 mL, from which one half contained ozonated VOO and the other half ozonated POO. Finally, two of the bottles of each type of oil were stored at 4 °C and the remaining two at room temperature (20 °C). All samples were stored in amber bottles inside boxes to avoid photooxidation of the samples. The physicochemical analyses and the evaluation of the antimicrobial activity were performed by triplicate on samples extracted directly from these bottles. In addition, the samples were analyzed at different times during the six-month storage period (0, 1, 10, 30, 90, and 180 days). Figure 1 contains a detailed illustration of the experimental design followed in this study.

### 2.3. Reagents

Chloroform (purity ≥ 99.8%), glacial acetic acid (purity ≥ 99%), and acetic acid (purity ≥ 99%) were purchased from Fisher Chemical (Hampton, NH, USA). Potassium iodide (saturated aqueous solution of 14 g/10 mL, purity 99%) was supplied by Thermo Scientific (Waltham, MA, USA). Sodium thiosulfate (aqueous solution of 0.01 M), starch, and phenolphthalein were obtained from Scharlau (Barcelona, Spain). Diethyl ether (purity 100%) and cyclohexane (purity ≥ 99.8%) were purchased from VWR Chemicals (Radnor, PA, USA). Ethanol (purity 96%) was supplied by Productos OPPAC S.A. (Madrid, Spain). Potassium hydroxide was obtained from Fisher Chemical (Hampton, NH, USA). The Wijs reagent was purchased from VWR Chemicals (Radnor, PA, USA).

### 2.4. Quality Parameters

All physicochemical analyses were performed by triplicate 1, 10, 30, 90, and 180 days after the ozonation treatment. Non-ozonated olive oil samples from both pomace and virgin olive oil were also analyzed as control treatments. Peroxide index (PI), acidity index (AI), and iodine value (IV) were carried out according to the methodologies described by the International Olive Council [25].

In order to calculate the PI, 0.5 g of the oil sample was weighed into a flask and 10 mL of chloroform and 15 mL of acetic acid were added to dissolve the oil. Then, 1 mL of a saturated solution (14 g/10 mL) of potassium iodide was added and it was left in the dark for 5 min. After that, 75 mL of distilled water was added and the iodine released was titrated using a 0.5 M sodium thiosulphate solution with starch as an indicator.

The acidity value was calculated by weighting 0.5 g of the oil sample and dissolving it in a 50 mL mixture of diethyl ether and ethanol at 95% (*v*/*v*), previously neutralized. The dissolution was titrated with a 0.1 M potassium hydroxide solution until the phenolphthalein indicator turned to a pink colour for at least 10 s.

The determination of the iodine value was carried out by weighing 0.2 g of the sample into a 500 mL flask and adding 20 mL of a solvent previously prepared with equal volumes of cyclohexane and acetic acid. Then, 25 mL of the Wijs reagent was added and the mixture was left in the dark for 2 h. After the time had passed, 20 mL of a potassium iodide solution (100 g/L) and 150 mL of distilled water were added. The solution was titrated with a 0.01 M sodium thiosulphate solution using starch until the blue colour disappeared. Viscosity (V) measurements were driven in an HAAKE K15 Rotovisco 1 viscosimeter using a Z20 DIN rotor purchased from Thermo Fisher Scientific (Waltham, MA, USA). Measurements were also taken by triplicate by using 7 mL of oil in a continuous ramp from 0 to 500 Pa at 25 °C.

### 2.5. Antimicrobial Activity Assays

In order to assess the antimicrobial activity of the ozonated olive oils, we followed the agar well diffusion method procedure described by the Spanish Society of Infectious Diseases and Clinical Microbiology for studying antimicrobial sensitivity [26]. All bacterial strains were purchased from the Spanish Type Culture Collection (STCC). The bacterial strains *Escherichia coli* (STCC 45), *Pseudomonas aeruginosa* (STCC109), and *Staphylococcus aureus* (STCC 239) were preserved in cryovials with glycerol at −85 °C. All bacteria were cultured on Tryptic Soy Agar (TSA) purchased from Condalab (Madrid, Spain) at 37 °C for 24 h. In order to adjust optical density (OD), a calibration curve correlating absorbance values (OD 600 nm) with specific bacterial concentrations (CFU/mL) was prepared. Dilutions were made, and absorbance was measured with a bench-top spectrophotometer Multiskan GO purchased byThermo Fisher Scientific (Vantaa, Finland) to create this curve. The microbial inoculum size was adjusted to 1 × 10^8^ CFU/mL for all assays. The agar well diffusion method was performed by adding 100 µL of ozonated olive oil in each well. The ozonated olive oils were mixed (2% *v*/*v*) with Tween 80 (Scharlab, Barcelona, Spain) to enhance the dispersion of the oil in the agar. The plates were incubated for 24 h at 37 °C, and inhibition zones were measured. The negative control consisted of a mixture of non-ozonated olive oil and Tween 80 (2% *v*/*v*) to verify that none of the components exhibited any bactericidal activity against the tested pathogens. The positive control consisted of bacteria growing on TSA plates without any antimicrobial agent, allowing us to confirm that any observed antimicrobial effects were specifically due to the ozonated oils.

### 2.6. Statistical Analysis

All analytical measurements in this study were performed by triplicate. The data obtained were tabulated and presented as means ± standard deviation (SD). The results were analyzed using Statgraphics v.19 (Alexandria, VA, USA). One-way analysis of variance (ANOVA) test and Tukey’s HSD test were conducted to determine significant differences between treatments and olive oil samples at *p*-value of 0.05 (*p* < 0.05). Then, Spearman’s rank correlation test was applied to highlight significant correlations. The coefficient ranges from −1 to 1, where values closer to 1 or −1 indicate strong positive or negative monotonic relationships, respectively. A value of 0 suggests no significant correlation. Statistical significance set at *p* < 0.05. These analyses were performed in RStudio (Version 2024.09.1-394).

## 3. Results and Discussion

### 3.1. Effect of Storage Conditions on Quality Parameters of Ozonated Olive Oils

The stability study carried out showed (Table 1) an upward trend in the peroxide index of both oils regardless of the storage temperature (4 °C and 20 °C) up to day 30. In both types of oil, the maximum values were reached in the samples stored at room temperature after 30 days, these values being 741.44 mEq O_2_/kg for VOO and 1067.23 mEq O_2_/kg for POO. Ozonated POO showed higher PI values than VOO from the beginning (day 0) to the end (day 180), since its starting peroxide value was significantly (*p* < 0.05) higher (119.53 mEq O_2_/kg against 13.03 mEq O_2_/kg). From day 90 onwards, the trend in the peroxide index of both oils was downward. However, the drop was not the same for the oils stored at room temperature as for the oils stored at 4 °C. The PI of VOO stored at 20 °C suffered a decrease of 18.50% from day 30 to day 180, while that of POO was 15.05%. However, the PI of both oils stored at 4 °C remained practically constant, decreasing only by 6.37% for VOO and 7.95% for POO. The observed trends are concurrent with the results previously published by [20,21,23], where it can also be seen how ozonized oils reach a maximum PI value and then, from day 90 [20] or even 30 [21,23], it suffers a drop. In accordance with our results, refs. [22,23] observed that the decrease in the PI is smaller at low temperatures (4 °C) and practically inexistent at freezing temperatures (−20 °C). This is due to the thermosensitivity of the hydroperoxides and ozonides formed during ozonation. Other studies [20,21] demonstrated that at high temperatures (37 °C–40 °C), the peroxide index drops drastically, even suffering a decrease of practically 100% after 90 days [23]. This suggests that ozonated oils stored at high temperatures are more vulnerable to suffering a decay in the peroxide index.

These results showed that the PI decreases as ozonides and hydroperoxides disappear, while carboxylic acids are formed. The formation of carboxylic acids led to increased acidity in all oils (Table 1) over time. For both types of oils, the maximum acidity value was reached at 180 days. Furthermore, and in agreement with the results obtained for the PI, greater values were reached in the samples stored at 20 °C; these values were 3.80 mg KOH/g for VOO and 6.32 mg KOH/g for POO. As expected, POO maintained higher acidity values (t = 0 days, 3.03 mg KOH/g) than VOO (t = 0 days, 1.08 mg KOH/g) from the beginning of the study. The steady increase in acidity from day 0 to day 180 for both types of oils, VOO (71.57%) and POO (52.05%), is consistent with the increase observed in other studies [16,18]. This increase is due to the decomposition of peroxide species and mainly due to the formation of carboxylic acids as by-products of the ozonolysis reaction. Since this degradation is favoured at higher temperatures, both in POO and VOO, the acidity was slightly higher in oils stored at 20 °C. Although the increase in AI was lower, 68.23% for VOO and 48.03% for POO, in ozonated oils stored at 4 °C, other authors such as Moureu et al. [23] observed an even lower increase in ozonated oils stored at 4 °C and was practically nonexistent in those stored at freezing temperature.

Carboxylic acids can be formed from ozonation by-products such as aldehydes, either because they are produced during the breaking of the double bond (C=C), or by thermal oxidation of unsaturated fatty acids. What has not been studied so far is the relationship that this has with the iodine value. Because some unsaturations of the ozonated oils are still present during thermal degradation of the ozonides (Table 1), the acid number continues to increase steadily over the 180 days of storage. Both oils started from a similar iodine value (94.33 g I/100 g for VOO and 87.08 g I/100 g for POO) due to the fact that both oils come from the same olives and, therefore, they have a very similar lipid profile. Therefore, the iodine index does not show significant (*p* < 0.05) differences between the oils. These differences are also inexistent between the same oil type stored at different temperatures. Although this consumption of double bonds has a gradual trend from day 10 to day 180, a drastic drop (40.66% for VOO and 39.28% for POO) was clearly observed from the initial value to the day after ozone treatment. This reveals interesting information about ozone dynamics. After analyzing these trends, we can affirm, like other authors [18,27], that the ozonolysis reaction and the consequent formation of ozonides takes place during the first hours after O_3_ treatment. Other authors suggested [3] that this reaction was more gradual and could last up to 4 days. However, it was clearly shown (Table 1) that the decrease in the IV was smoother after 10 days (9.50% for VOO and 10.77% for POO) and the following (30, 90, 180 days) number of days.

The evolution observed in viscosity agrees with the results obtained in other quality parameters. As expected from the PI results, viscosity reached its maximum after 30 days regardless of the type of oil and storage temperature. Additionally, these values suffered a slight decay from day 30 until 180; and this decrease was more pronounced in ozonated oils stored at room temperature. Viscosity is increased by the formation of molecules with a higher molecular weight [28], that is, to the formation of ozonides and hydroperoxides. Therefore, as with PI, when these heavy compounds degrade, viscosity decreases. And, as mentioned before, this reaction is accelerated at higher temperatures.

Previous studies have characterized ozonated vegetable oils and analyzed the physicochemical changes induced by ozonation. Guerra-Blanco et al. [29] observed that, regardless of the oil type (grapeseed, avocado, sunflower, or olive), the accumulation of ozonides reaches a threshold within a few hours of ozonation. This occurs because all C=C double bonds are consumed, stopping the ozonolysis reaction. Our findings complement this dynamic, as shown in Table 1, where the peroxide index (PI) reaches stability by day 30 and then declines due to the degradation of peroxides over time. While this trend is consistent across different oils, previous studies [30] reported significant variations in PI, AI, IV, and V among ozonated olive, hemp, and coconut oils. For example, under the same ozonation treatment, hemp oil exhibited a higher peroxide index (208 mEq O_2_/kg) compared to coconut (68 mEq O_2_/kg) and olive oil (108 mEq O_2_/kg). In contrast, another study [17] reported much higher PI values for ozonated olive (3110 mEq O_2_/kg) and sunflower oil (3520 mEq O_2_/kg), along with significantly higher acidity indices (9.93 KOH/g and 28 KOH/g, respectively), compared to our results. Unlike previous studies, which analyzed changes only after 24 h from ozonation, our study examines the evolution of these parameters over time and under different storage conditions. The trends observed for VOO and POO at 4 °C and 20 °C suggest that similar patterns may occur in other vegetable oils. However, further research is needed to confirm whether, regardless of oil type or ozonation treatment, PI, IV, and V consistently decrease over time while AI increases.

### 3.2. Effect of Storage Conditions on Antimicrobial Efficiency of Ozonated Olive Oils

**Figure 2:**
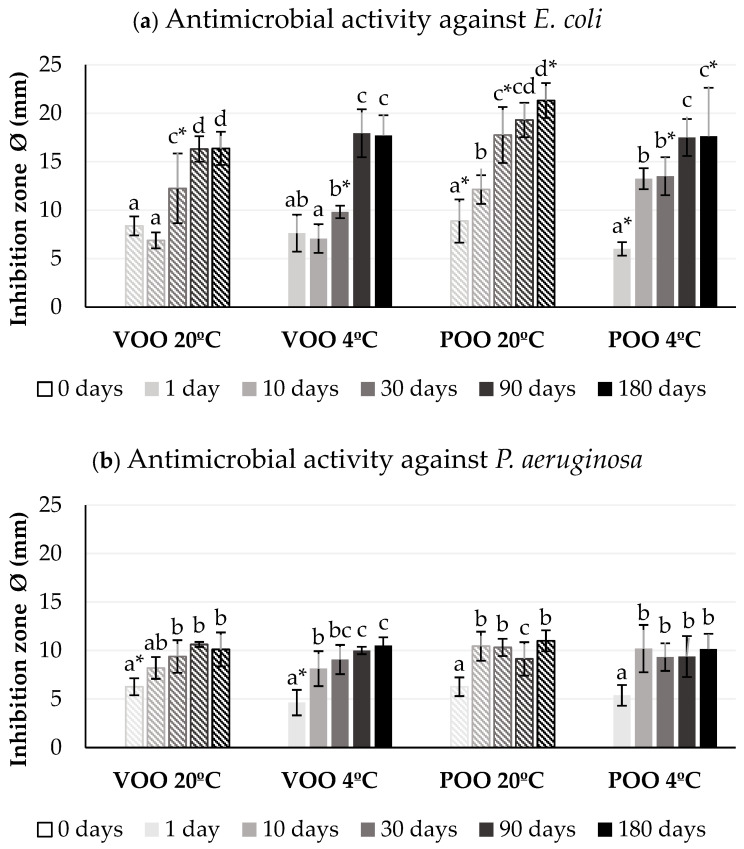

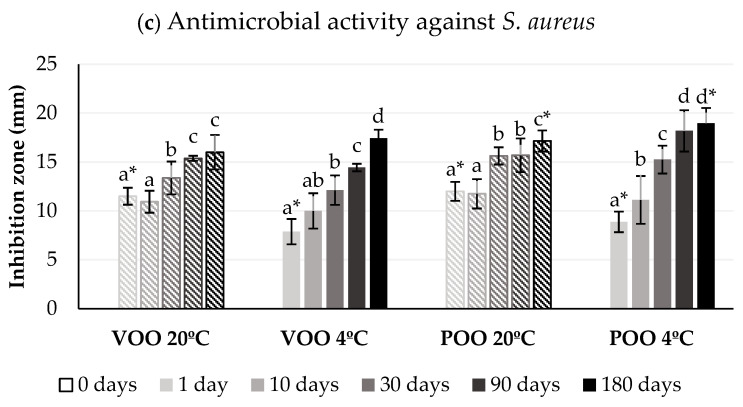
Evolution of antimicrobial activity against (**a**) *E. coli*, (**b**) *P. aeruginosa*, and (**c**) *S. aureus* of ozonated virgin olive oil (VOO) and pomace olive oil (POO) stored at different temperatures. Vertical bars represent standard deviations for mean values. Different lower-case letters indicate significant difference (*p* < 0.05) among storage days for same sample. Statistical differences (*p* < 0.05) between same oil types stored at different temperatures indicated with asterisk (*).

The storage conditions, in terms of time and temperature, had an observable effect on the antimicrobial activity of ozonated olive oils (Figure 2). First of all, it is worth mentioning that the inhibition diameters observed in *S. aureus* were higher than those in *E. coli* and *P. aeruginosa*, in terms of mean values (*E. coli*, Ø = 13.88 mm ± 0.49; *P. aeruginosa*, Ø = 9.41 mm ± 0.23; S. aureus Ø = 14.49 mm ± 0.36) regardless of the type of oil. Ozonides are able to oxidize glycolipids, glycoproteins, enzymes, and genomic material in microorganisms, with Gram-positive bacteria (*S. aureus*) being more susceptible to ozonated oils than Gram-negative bacteria (*E. coli*, *P. aeruginosa*) due to the structural differences in their cell wall structures [17]. Secondly, it is worth noting that there were no statistically significant differences (*p* < 0.05) between the two types of ozonated oil, except in the case of *E. coli* (Figure 2a), where the ozonated pomace oil was more effective when stored at 20 °C than ozonated virgin olive oil.

Regarding the differences between oils stored at different temperatures, it should be noted (Figure 2b) that in *P. aeruginosa*, the bactericidal capacity of the ozonated oils did not change regardless of whether they were stored at 20 °C or 4 °C, nor did it change throughout the days. This indifference in the antimicrobial activity coincides with the results published by Moureau et al. [23] for *Streptococcus uberis*, where they found that the antimicrobial activity was not altered in spite of the structural changes that the oils underwent at different storage temperatures (−20 °C, 4 °C, 20 °C, and 37 °C). However, in this study, we were able to demonstrate that this trend is not maintained for all microorganisms, since the effectiveness of ozonated oils against *E. coli* and *S. aureus* was altered as the days went by and the storage temperature.

As can be seen for *E. coli* (Figure 2a) and for *S. aureus* (Figure 2c), with the progression of days, the antimicrobial activity increased gradually, with this increase being more pronounced in *E. coli*. The highest antimicrobial activity values were achieved after 180 days in both types of oil, the highest being in POO preserved at 20 °C for *E. coli* (Ø = 21.31 ± 4.01 mm) and VOO preserved at 4 °C for *S. aureus* (Ø = 18.97 ± 1.46 mm). There are significant differences (*p* < 0.05) between all treatments after day 30 and the differences between temperatures depend on the microorganism. If we consider the mean values of antimicrobial activity against *E. coli*, we can observe that the oils preserved at 20 °C have a slight but not significant antimicrobial activity (Ø = 13.94 ± 0.71 mm) than those preserved at 4 °C (Ø = 13.43 ± 1.48 mm). However, the effect of the temperature observed for *S. aureus* suggested the contrary, with the activity of the oils stored at 4 °C (Ø = 13.96 ± 1.09 mm) being higher than those preserved at 20 °C (Ø = 12.08 ± 2.71 mm).

A possible explanation for the increase in antimicrobial activity in *E. coli* and *S. aureus* throughout the days could lie in the physicochemical results obtained. The acidification of the ozonated oils over time (Table 1) may have increased the antimicrobial activity, indicating that these microorganisms are more sensitive to acidity than *P. aeruginosa*. On the other hand, the slight differences observed in storage temperature for the *S. aureus* strain may be due to the chemical reactivity of ozone at low temperatures [31]. It is known that primary ozonide species, such as 1,2,3-trioxolane rings, are more unstable and can be broken at low temperatures. This cleavage generates the formation of zwitterionic carbonyl oxide and a carbonyl compound (Criegee intermediates) [4]. They can interact and recombine to deliver the 1,2,4-isomer, known as the second stable ozonide (SOZ). Therefore, a possible explanation would be that oils ozonated at low temperatures have a higher amount of these compounds and that *S. aureus* is more sensitive to them.

**Figure 3:**
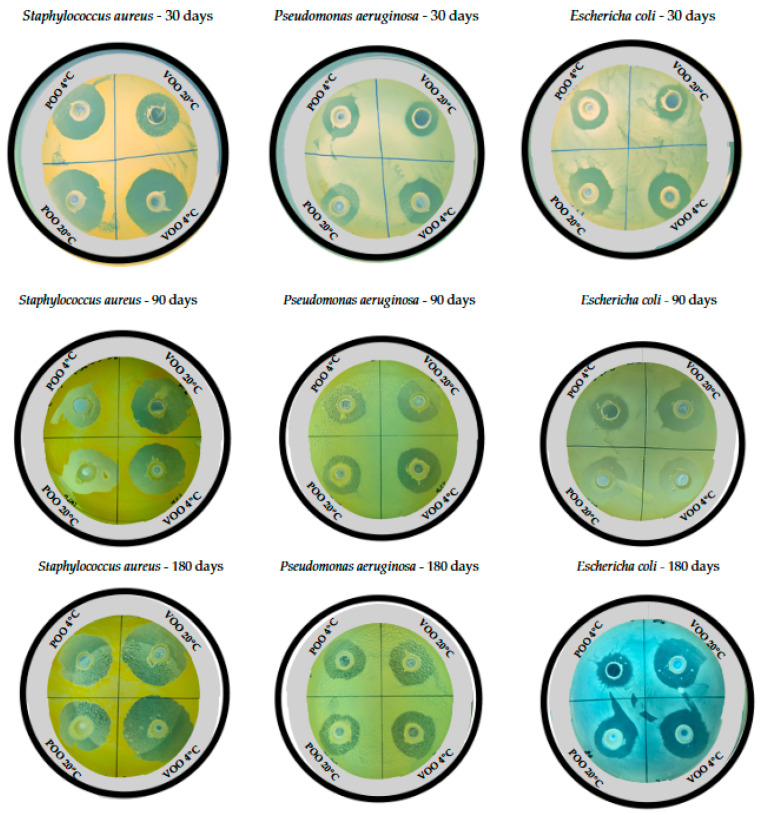
Image of inhibition zones obtained with agar well diffusion method for *S. aureus*, *P. aeruginosa*, and *E. coli* after testing ozonated virgin olive oil (VOO) and pomace olive oil (POO) samples stored at 4 °C and 20 °C for 30, 90, and 180 days.

Evaluating the antimicrobial activity of ozonated vegetable oils is challenging due to their oily nature, viscosity, and turbidity. Some studies [17] found some difficulties measuring the MIC due to interferences in optical density. A previous study [23] showed that the MIC of ozonated oils remained stable (5–10 mg/mL) over a year, regardless of the storage conditions. However, our results indicate that this is not reproducible for all microorganisms, and that the antimicrobial potency of ozonated oils can change over time, as reflected in the inhibition zones (Figure 3). Similarly to Balea et al. [30], we found that the antimicrobial effectiveness varies depending on the bacterial strain. Balea et al. observed higher inhibition halos against *P. aeruginosa* (14 mm) than *E. coli* (12 mm) or *S. aureus* (10 mm). Our findings suggest that ozonated olive and pomace oils are more effective against *S. aureus* (14 mm), which aligns with previous results [32], where ozonated sunflower and olive oils showed the lowest MIC (4.5 mg/mL) for *S. aureus*. This variability also depends on the strain, as [13] found different MICs for various *E. coli* strains. Importantly, neither we nor other studies [17,32] observed any antimicrobial activity in non-ozonated oils.

### 3.3. Correlation Among Storage Variables, Quality Parameters, and Antimicrobial Activity

**Figure 4:**
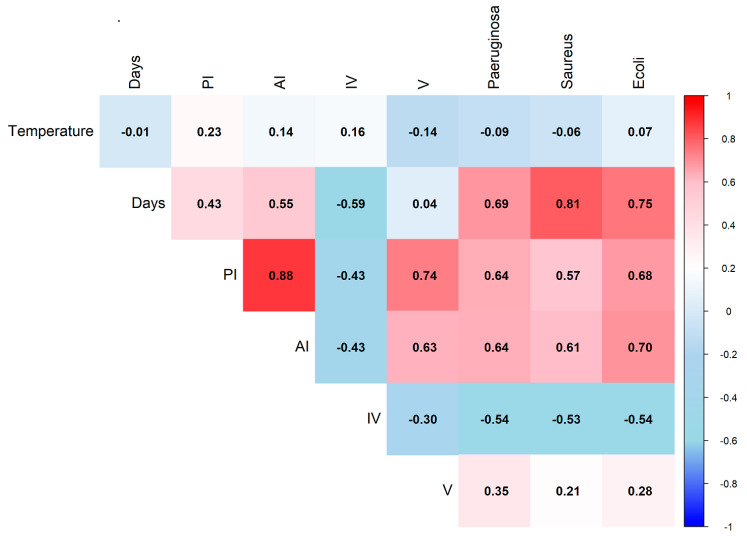
Heatmap of Spearman correlation coefficients for storage conditions variables of temperature and days; quality parameters of peroxide index (PI), acidity index (AI), iodine value (IV), and viscosity (V); and antimicrobial activity against *S. aureus*, *P. aeruginosa*, and *E. coli* for both ozonated virgin (VOO) and pomace (POO) olive oils.

The storage time of the ozonated oils led to significant differences (*p* < 0.05) both in the physicochemical parameters and in the antimicrobial activity of the two oils (VOO and POO). Moreover, both ozonated virgin and pomace olive oils can be stored at room temperature without compromising their antimicrobial efficacy (Figure 4). Consequently, the elimination of refrigeration requirements can significantly optimize storage and transportation logistics, leading to a reduction in associated costs.

Based on the Spearman correlation test, a strong positive relationship (ρ = 0.88, *p* < 0.05) was observed between peroxide index (PI) and acidity index (AI), indicating that the formation of carboxylic acids is closely linked to the decomposition of hydroperoxides and ozonides. Additionally, PI and AI showed a significant positive correlation with antimicrobial activity across all tested strains, with *S. aureus* exhibiting the strongest association (ρ = 0.81, *p* < 0.05). A positive and robust correlation was also detected between storage time and antimicrobial activity, as well as between storage time and both PI and AI. This suggests that the progressive physicochemical changes in the oils during storage, particularly the increase in acidity and the breakdown of unstable ozonation products, enhance antimicrobial efficacy over time. Several studies have reported findings consistent with our results. For instance, a study demonstrated [33] that higher acidity enhances antimicrobial activity. Additionally, the same study found that *Staphylococcus aureus* was more susceptible to ozonated oils compared to Gram-negative bacteria, aligning with our observations.

Furthermore, a negative correlation was found among PI, AI, and iodine value (IV), confirming that the consumption of double bonds is inversely related to the accumulation of ozonides and hydroperoxides. Viscosity (V) also exhibited a positive and strong correlation with PI (ρ = 0.74, *p* < 0.05), suggesting that the formation of high-molecular-weight compounds contributes to viscosity changes. These findings emphasize the dynamic interplay between ozonation-induced oxidative changes and antimicrobial activity, further highlighting the influence of storage time on the bioactive properties of ozonated oils.

To sum up, over time, ozonated oils suffered an initial increase in peroxide values, reaching their maximum at day 30, followed by a decline, while acidity values consistently rise, peaking at day 180. Notably, oils stored at 20 °C showed slightly higher PI and AI values compared to those stored at 4 °C. These changes were more pronounced in pomace olive oil (POO), which consistently showed higher PI and AI values than virgin olive oil (VOO). Despite these physicochemical changes, the antimicrobial activity of ozonated olive oils remained effective over prolonged storage periods (6 months). Antimicrobial activity against *S. aureus* was stronger than against *E. coli* and *P. aeruginosa*, with the highest inhibition diameters observed after 180 days of storage (18.97 ± 1.46 mm, VOO stored at 4 °C). A strong positive correlation (ρ = 0.81, *p* < 0.05) was found among PI, AI, and antimicrobial efficacy, particularly against *S. aureus*.

## 4. Conclusions

This study highlights the significant impact of storage time on the physicochemical properties and antimicrobial efficacy of ozonated virgin and pomace olive oils. These findings confirm that ozonated oils can be stored at room temperature without compromising quality or antimicrobial activity, with potential cost savings in storage and transportation. Overall, the dynamic interplay between physicochemical changes and bioactivity underscores the potential of ozonated olive oils as effective antimicrobial agents, making them valuable for food preservation. Moreover, pomace, a by-product of olive oil processing, adds further potential for such applications. Future research should evaluate how ozonated vegetable oils perform in food preservation, particularly in high-risk products like dairy, meat, and fresh produce.

## Figures and Tables

**Table 1:** Evolution between 0 and 180 days of main quality parameters (peroxide index, acidity index, iodine value, and viscosity) of ozonated virgin olive oil (VOO) and pomace olive oil (POO) stored at different temperatures (4 °C and 20 °C).

***Peroxide Index Values***						
**Sample**	**0 days**	**1 day**	**10 days**	**30 days**	**90 days**	**180 days**
VOO 20 °C	13.03 ± 5.92 ^Aa^	590.43 ± 40.03 ^Ba^	634.87 ± 41.23 ^Ba^	741.44 ± 32.16 ^Ca^	689.73 ± 47.81 ^Ca^	604.23 ± 10.09 ^Da^
VOO 4 °C	13.03 ± 5.92 ^Aa^	350.25 ± 36.64 ^Bb^	430.21 ± 21.16 ^Cb^	534.78 ± 49.62 ^Db^	524.84 ± 19.01 ^Eb^	500.68 ± 35.21 ^Eb^
POO 20 °C	119.53 ± 9.22 ^Aa^	875.19 ± 74.35 ^Ba^	1032.74 ± 76.73 ^Ca^	1053.40 ± 70.97 ^Ca^	1036.07 ± 83.50 ^Ca^	906.62 ± 14.69 ^Da^
POO 4 °C	119.53 ± 9.22 ^Aa^	674.33 ± 54.31 ^Bb^	868.74 ± 70.23 ^Cb^	1067.23 ± 56.56 ^Da^	1004.78 ± 43.90 ^Da^	982.36 ± 23.36 ^Db^
***Acidity Index Values***						
**Sample**	**0 days**	**1 day**	**10 days**	**30 days**	**90 days**	**180 days**
VOO 20 °C	1.08 ± 0.02 ^Aa^	2.38 ± 0.07 ^Ba^	2.93 ± 0.18 ^Ca^	3.49 ± 0.14 ^Da^	3.33 ± 0.19 ^Da^	3.80 ± 0.05 ^Ea^
VOO 4 °C	1.08 ± 0.02 ^Aa^	1.62 ± 0.15 ^Bb^	2.23 ± 0.40 ^Cb^	3.08 ± 0.21 ^Db^	3.18 ± 0.12 ^Da^	3.40 ± 0.07 ^Eb^
POO 20 °C	3.03 ± 0.26 ^Aa^	4.67 ± 0.34 ^Ba^	5.43 ± 0.28 ^Ca^	5.81 ± 0.22 ^Da^	6.14 ± 0.22 ^DEa^	6.32 ± 0.14 ^Ea^
POO 4 °C	3.03 ± 0.26 ^Aa^	3.55 ± 0.46 ^Bb^	4.25 ± 0.36 ^Cb^	5.02 ± 0.31 ^Db^	5.72 ± 0.26 ^Eb^	5.83 ± 0.18 ^Eb^
***Iodine Values***						
**Sample**	**0 days**	**1 day**	**10 days**	**30 days**	**90 days**	**180 days**
VOO 20 °C	94.33 ± 3.07 ^Aa^	55.99 ± 7.12 ^Ba^	50.67 ± 10.65 ^BCa^	47.93 ± 2.42 ^BCa^	48.75 ± 4.13 ^BCa^	44.37 ± 2.90 ^Ca^
VOO 4 °C	94.33 ± 3.07 ^Aa^	54.62 ± 8.27 ^Ba^	44.04 ± 4.25 ^Ca^	43.38 ± 5.20 ^Ca^	41.26 ± 5.19 ^Ca^	36.46 ± 5.71 ^Ca^
POO 20 °C	87.08 ± 2.73 ^Aa^	58.33 ± 3.81 ^Ba^	48.10 ± 6.45 ^Ca^	41.59 ± 2.60 ^CDa^	41.20 ± 2.47 ^CDa^	37.38 ± 3.19 ^Da^
POO 4 °C	87.08 ± 2.73 ^Aa^	52.87 ± 4.75 ^Ba^	47.21 ± 4.20 ^Ca^	48.74 ± 5.87 ^Ca^	45.11 ± 2.09 ^Ca^	39.81 ± 0.94 ^Ca^
***Viscosity Values***						
**Sample**	**0 days**	**1 day**	**10 days**	**30 days**	**90 days**	**180 days**
VOO 20 °C	60.22 ± 0.77 ^Aa^	343.20 ± 14.80 ^Ba^	335.28 ± 2.96 ^Ba^	318.56 ± 0.16 ^Ca^	290.99 ± 0.77 ^Da^	232.19 ± 9.05 ^Ea^
VOO 4 °C	60.22 ± 0.77 ^Aa^	358.45 ± 3.30 ^Ba^	365.26 ± 18.89 ^Ba^	372.82 ± 6.56 ^Bb^	355.83 ± 24.98 ^Bb^	309.52 ± 2.80 ^Cb^
POO 20 °C	64.90 ± 0.76 ^Aa^	665.75 ± 7.92 ^Ba^	605.40 ± 0.12 ^Ca^	573.35 ± 1.23 ^Da^	445.60 ± 38.46 ^Ea^	401.42 ± 2.83 ^Fa^
POO 4 °C	64.90 ± 0.76 ^Aa^	627.75 ± 28.14 ^Ba^	597.66 ± 3.15 ^Bb^	651.77 ± 0.32 ^Cb^	624.18 ± 5.72 ^BDb^	602.45 ± 3.12 ^Db^

Results are expressed as mean values ± standard deviation (s.d.). Different upper-case letters indicate statistical differences (*p* < 0.05) among days for same sample and different lower-case letters indicate statistical differences (*p* < 0.05) between storage temperatures for same oil type.

## Data Availability

The original contributions presented in this study are included in the article. Further inquiries can be directed to the corresponding author.
